# Pathogenic Th2 Cytokine Profile Skewing by IFN-γ-Responding Vitiligo Fibroblasts via CCL2/CCL8

**DOI:** 10.3390/cells12020217

**Published:** 2023-01-04

**Authors:** Rong Jin, Miaoni Zhou, Fuquan Lin, Wen Xu, Aie Xu

**Affiliations:** Department of Dermatology, Hangzhou Third People’s Hospital, Hangzhou 310009, China

**Keywords:** vitiligo, fibroblast, T cells, CCL2, JAK

## Abstract

Purpose: Vitiligo is a T cell-mediated skin depigmentation disease. Though treatments arresting disease progression and inducing repigmentation are available, the efficacy of these options is often limited and poorly sustained. How stromal signals contribute to the interferon-γ-dominant skin niches is unclear. This study aims to determine how fibroblasts participate in the IFN-γ-dominant vitiligo niche. Patients and methods: Mouse vitiligo models were established. Fibroblasts from control and vitiligo mice were extracted for RNA sequencing. In vitro IFN-γ stimulation was performed to verify the JAK-STAT pathway by qPCR and Western blot. T cell polarization with chemokines was measured by flow cytometry. Protein levels in tissues were also examined by IHC. Results: The vitiligo mouse model recapitulates the human CD8-IFN-γ pathway. RNA sequencing revealed elevated chemokine CCL2 and CCL8 in vitiligo fibroblast, which may be regulated by the JAK-STAT signaling. Such phenomenon is verified by JAK inhibitor peficitinib in vitro. Moreover, CCL2 addition into the naïve T polarization system promoted type 2 cytokines secretion, which represents a hallmark of vitiligo lesions. Conclusion: Dermal fibroblasts, a principal constituent of skin structure, respond to IFN-γ by skewing T cells towards a type 2 cytokine profile via CCL2 and CCL8, which can be abrogated by JAK inhibitor peficitinib.

## 1. Introduction

Vitiligo is a T-cell-mediated depigmentation disorder characterized by melanocyte destruction, manifesting as white patches on the skin, affecting 0.5–2% of the population worldwide [1]. Though treatments arresting disease progression and inducing repigmentation are available, the efficacy of these options is often limited and poorly sustained [2].

Fibroblasts constitute a major component of the dermal compartment, with mounting evidence indicating their role in inflammatory skin diseases [3,4,5]. Though they have been recognized for regulating epidermal pigmentation [6,7], their contribution to the interaction of immune cells and stromal signals has been rather descriptive and not precisely defined [8]. Chemokine secretion represents a central mechanism fibroblasts employed to manipulate immune crosstalk [9]. CCL2 and CCL8 are two CC chemokines capable of acting upon macrophages and type 2 T lymphocytes [10,11,12,13,14]. Though previous studies focused on CXCL9/CXCL10-induced interferon -γ from T cells [15], the pivotal cytokine shaping a vitiligo-prone milieu, the involvement of type 2 cytokine signatures has been recently suggested [16].

Recently, JAK inhibitor treatments, including ruxolitinib, tofacitinib, and baricitinib, showed dramatic repigmentation in patients [17]. The Janus kinase/signal transducer and activator of transcription (JAK/STAT) signaling pathway are central in cytokine production, including IFN-γ [18]. Nevertheless, inhibiting potential IFN-γ-producing T cells may not be the only mechanism accounting for the superior effect. This study proposed that IFN-γ activates JAK/STAT signaling in fibroblasts to express CCL2 and CCL8, ending in type 2 T cell attraction differentiation. Our results highlighted a new stromal-type 2 immune crosstalk pathway and provided an alternative rationale for JAK inhibitor treatment in vitiligo.

## 2. Materials and Methods

### 2.1. Mouse Vitiligo Model

All animal experiments complied with ethical regulations and were approved by the subcommittee on Research Animal Care of the Fourth Military Medical University. Adapted from what was previously described [3,19,20], C57BL/6J mice (female) were inoculated intradermally on the left flank with 2 × 10^5^ B16F10 cells on day 0 and treated with anti-CD4 mAb (Bio X cell, Lebanon, NH, USA) intraperitoneally on day 4 and 10, to eliminate regulatory T cells. Tumors were surgically excised on day 12. Spontaneous metastases were not observed with this B16F10 subline. Mice were monitored for the development of vitiligo 4 weeks post-surgery by harvesting tail ends for whole-mount immunostaining, and only mice that developed vitiligo (∼90%) were used for subsequent studies. Mice with vitiligo were subsequently enrolled in experiments. When more than one group was being compared, the initial group assignment was completed with the intent of matching the overall level of vitiligo between the groups. All procedures were approved and supervised by the subcommittee on Research Animal Care of the Fourth Military Medical University.

### 2.2. Immunofluorescence

Mouse skin tissue was fixed in 4% paraformaldehyde and frozen sections (10 µm) were fixed in ice-cold methanol for 30 min. Sections were blocked with blocking buffer containing 1% FBS, 1% BSA, and 0.3% Triton-X 100 for 1 h at room temperature, then stained with anti-CCL2 (Genetex, Alton Pkwy Irvine, CA, USA) and anti-CCL8 (Thermo, Waltham, MA, USA) primary antibodies overnight at 4 °C. Sections were washed three times in PBS and stained with goat anti-rabbit IgG (H + L) highly cross-adsorbed secondary antibody, Alexa Fluor Plus 488 (1:100, Invitrogen, Carlsbad, CA, USA) was incubated for 1 h protected from light. Nuclei were stained with DAPI (BD Biosciences, San Jose, CA, USA) for 5 min at room temperature and protected from light. After washing with PBS, they were observed under a confocal microscope (Leica, Wetzlar, Germany). For cell samples, after treatment in cell culture dishes, cells were washed three times with PBS and fixed in 4% paraformaldehyde at room temperature for 15 min after washing with PBS, cells were permeabilized with 0.1% Triton-X 100 (Beyotime, Nantong, China) for 10 min, blocked with immunofluorescent blocking solution (Beyotime, Nantong, China) for 30 min at 37 °C, washed again and stained with anti-8-OH-dG (Abcam, Cambridge, MA, USA) primary antibody at 4 °C overnight. The next day’s experiments were processed for the tissue samples.

### 2.3. ROS Staining

Collect the cells in fluorescent dish, and the cells were washed twice with serum-free medium. Configure 10 µM ROS probe--DCFH (Beyotime, Nantong, China) in 1 mL serum-free medium, incubate the cells at 37 °C for 30 min in the dark, and then wash twice with PBS. An additional 1 mL PBS was added to the fluorescent dish to visualize under a confocal microscope (Leica, Wetzlar, Germany).

### 2.4. RNA Extraction and Library Construction

Total RNA was isolated and purified using TRIzol reagent (Invitrogen, Carlsbad, CA, USA) following the manufacturer’s procedure. The RNA amount and purity of each sample were quantified using NanoDrop ND-1000 (NanoDrop, Wilmington, DE, USA). The RNA integrity was assessed by Bioanalyzer 2100 (Agilent, Santa Clara, CA, USA) with RIN number > 7.0, and confirmed by electrophoresis with denaturing agarose gel. We performed the 2 × 150 bp paired-end sequencing (PE150) on an Illumina Novaseq 6000 (LC-Biotechnology CO., Ltd., Hangzhou, China) following the vendor’s recommended protocol.

### 2.5. Bioinformatics Analysis of RNA-Sequencing

Verify sequence quality by removing values in Fastp software (https://github.com/OpenGene/fastp, accessed on 12 November 2021) for low-quality bases, default parameters containing adapter contamination, and unidentified bases. Reads were mapped to the reference genome using HISAT2 (https://ccb.jhu.edu/software/hisat2, accessed on 12 November 2021). Transcriptomes from all samples were then combined using gffcompare to reconstruct a comprehensive transcriptome. After final generation of the transcriptome, the expression levels of mRNAs were performed by StringTie (https://ccb.jhu.edu/software/stringtie, accessed on 12 November 2021). Differential genes between samples were analyzed by the R package edgeR (https://bioconductor.org/packages/release/bioc/html/edgeR.html, accessed on 12 November 2021) and a parametric F-test was performed to compare nested linear models (*p*-value < 0.05). Finally, genes were analyzed for GO and KEGG enrichment using the DAVID software.

### 2.6. mRNA Extraction Analysis by Quantitative PCR

Having followed the manufacturer’s instructions, total cellular RNA was extracted using the RNeasy Mini Kit (QIAGEN, Duesseldorf, Germany). An amount of 1 mg of total RNA was quantified spectrophotometrically and reverse transcribed into cDNA using the Transcriptor First Strand cDNA Synthesis Kit for qPCR (Roche, Basel, Switzerland). We used CFX96TM Real-Time System (Bio-Rad, Hercules, CA, USA) to perform quantitative polymerase chain reaction with Universal SYBR Green Master Mix (Roche, Basel, Switzerland). The mRNA levels of βactin were normalized to the control using CT values. The primer sequences are shown below:

*Ccl2* Forward, GAATCACCAGCAGCAAGTGTC;
*Ccl2* Reverse, CGGAGTTTGGGTTTGCTTGTC;
*Ccl8* Forward, TGGAGAGCTACACAAGAATCACC;
*Ccl8* Reverse, TGGTCCAGATGCTTCATGGAA;
*Jak2* Forward, CGAATGGTGTTTCTGATGTACC;
*Jak2* Reverse, GTCTCCTACTTCTCTTCGTACG;
*Stat1* Forward, CAGCTTGACTCAAAATTCCTGGA;
*Stat1* Reverse, TGAAGATTACGCTTGCTTTTCCT;
*β-actin* Forward, CCTTCCTGGGCATGGAGTC;
*β-actin* Reverse, TGATCTTCATTGTGCTGGGTG.

### 2.7. ELISA

After processing cells in groups in six-well plates, cell supernatants were collected and anti-CCL2 and anti-CCL8 levels were measured using ELISA kits according to the manufacturer’s instructions. Briefly, the cell supernatant was added to the microtiter plate, with duplicate wells set up for each group, and incubated at 37 °C for 1 h. The wells were washed four times with washing solution to remove unbound antibodies, 100 μL of streptavidin–HRP was added to each well, incubated for 1 h at 400 rpm on a room temperature shaker, washed again, and then 100 μL of tetramethylbenzidine, a substrate solution that reacts with HRP, was added to the wells, which were removed, incubated for approximately 10 min protected from light and then absorbance at 450 nm was measured using a microplate spectrophotometer (Molecular Devices, San Jose, CA, USA).

### 2.8. Western Blotting

Cells were lysed in RIPA buffer (Beyotime, Nantong, China) supplemented with phenylmethylsulfonyl fluoride (Beyotime, Nantong, China). Proteins were then separated by 15% SDS-PAGE gel and transferred onto the PVDF membrane (Millipore, Billerica, MA, USA). β-actin (Abcam, Cambridge, MA, USA), JAK2 (Abcam, Cambridge, MA, USA), STAT1 (Abcam, Cambridge, MA, USA), IFN-γ (Abcam, Cambridge, MA, USA), IL-4 (Abcam, Cambridge, MA, USA), S100A4 (Abcam, Cambridge, MA, USA), p-JAK2 (Abcam, Cambridge, MA, USA) and p-STAT1 (Abcam, Cambridge, MA, USA) primary antibodies and fluorescent dye-labeled secondary antibody were used. The density of protein bands was measured by an infrared imaging system (LI-COR, Lincoln, NE, USA).

### 2.9. Immunohistochemistry

Mouse tail skin tissue was fixed in 4% paraformaldehyde and then paraffin was embedded. Wax blocks were sectioned (4 μm) and dewaxed to water, antigen was repaired, and then closed in goat serum to block non-specific binding. Sections were incubated with antibodies against CCL2 (Genetex, Alton Pkwy Irvine, CA, USA) and CCL8 (Thermo, Waltham, MA, USA) diluted at a ratio of 1:100 overnight at 4 °C. Secondary antibodies were horseradish peroxidase-labeled rabbit anti-goat IgG (Invitrogen, Carlsbad, CA, USA), incubated for 1 h at 37 °C in a wet box protected from light, hematoxylin re-stained, and sealed in neutral gum before incubation in Olympus BX50 (Olympus, Tokyo, Japan) microscope.

### 2.10. T Cell Differentiation

Treat C57BL/6 mouse spleen tissue, crush on a cell filter to obtain a single cell suspension, and lyse erythrocytes. Naïve CD4+ T cells were sorted according to the MagniSort™Mouse CD4 Naïve T cell Enrichment Kit supplier time instructions (Invitrogen, Carlsbad, CA, USA).

For all T cell cultures, RPMI 1640 (Cytiva, Marlborough, MA, USA) medium was supplemented with 10% FBS (GIBCO, Grand Island, NY, USA), 10 mM HEPES buffer (GIBCO, Grand Island, NY, USA), 55 mM 2-ME (GIBCO, Grand Island, NY, USA), antibiotic–antifungal (GIBCO, Grand Island, NY, USA) and 2 mM L -glutamine (GIBCO, Grand Island, NY, USA), 1 mM sodium pyruvate (GIBCO, Grand Island, NY, USA) and MEM non-essential amino acids (GIBCO, Grand Island, NY, USA). About 100,000 cells were added to 96-well plates coated with anti-mouse CD3 (2.5 mg/mL, BD Pharmingen, San Diego, CA, USA) and soluble anti-mouse CD28 (2 mg/mL, BD Pharmingen, San Diego, CA, USA). T cells were cultured with CCL2 or CCL8 (MedChem Express, Monmouth Junction, NJ, USA).

### 2.11. Flow Cytometry and Intracellular Cytokine Staining

T cells were collected after 72 h of polarization and activated for 4 h using the cell stimulation cocktail (along with protein transport inhibitors) (Invitrogen, Carlsbad, CA, USA). Using the BD Fixation/Permeabilization Kit (BD Biosciences, San Jose, CA, USA), cells were fixed and permeabilized before being labeled with fluorescent IL-4 antibodies. Using FlowJo, data from a BD LSRFortessa flow cytometer were examined.

### 2.12. Statistical Analysis

All data were statistically analyzed using Graph Pad Prism 9 (version 9.0) and expressed as mean ± SEM. Comparisons were tested using the *t*-test, the two-way ANOVA, and the Šídák multiple comparisons test. Expressed as probability values, when *p* ≤ 0.05 was considered statistically different, ns, not statistically significant.

## 3. Results

### 3.1. The CD8-Mediated Mouse Vitiligo Model Features Human Vitiligo Hallmarks

**Figure 1:**
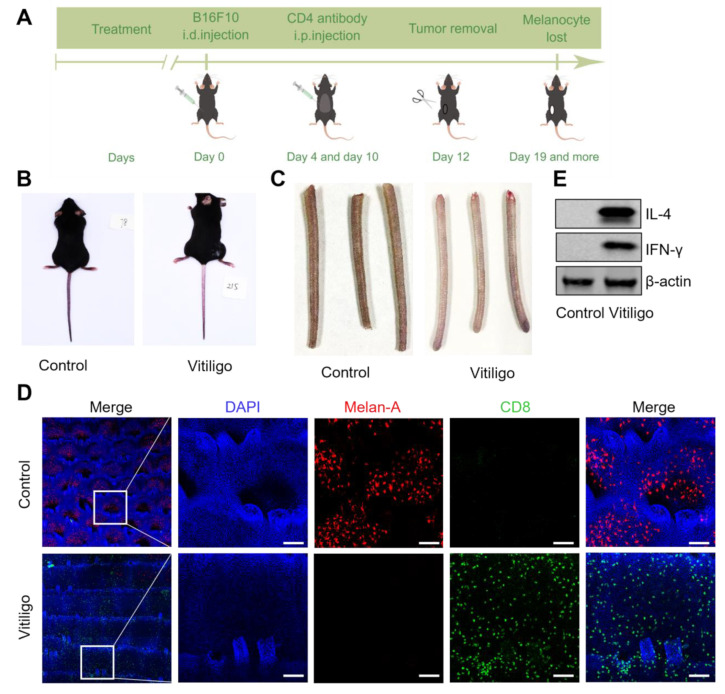
The CD8−mediated mouse vitiligo model features human vitiligo hallmarks. (**A**) Schematic diagram of the B16−induced vitiligo−like rat model. (**B**) Representative pictures of autoimmune vitiligo in mice after adoptive cell transfer showed remarkable depigmentation in tails (*n* = 3). (**C**) Representative enlarged tail views from mice in (**B**) (*n* = 3). (**D**) Representative immunofluorescent images suggested decreased melanocytes (red, stained with Melan−A) density and significant CD8+ T cell (green) infiltration from tails in (**B**) (*n* = 3), scale bars = 50 μm. (**E**) Representative Western blots showed elevated IFN−γ and IL−4 in vitiligo skin compared with control (*n* = 3).

Vitiligo pathogenesis is a complex interplay between melanocyte defects, environmental factors, and dysregulated immune niches. Such complexity hinders the clarification of immune–stromal crosstalk as most mouse vitiligo models cannot adequately capture the immune hallmarks in human vitiligo lesions. We established a model adopting melanoma-targeted immune responses as a strategy to recapitulate the vicious immune system in the skin. We injected B16 melanoma cells into C57BL/6J mice, followed by regulatory T cell depletion and excision of the established tumor (Figure 1A). After 3 months, we observed remarkable depigmentation, especially in tails (Figure 1B). Enlarged views further confirmed whiter tails in the vitiligo group compared with the control group (Figure 1C). In both human and this mouse model, CD8+ T cells secret IFN-γ to destroy melanocytes, resulting in depigmentation [21,22,23]. We thereby examined CD8+ T cells and melanocytes through immunofluorescence. Fluorescent images showed drastically decreased melanocyte density and significant CD8+ T cell infiltration (Figure 1D). Consistently, Western blot confirmed elevated IFN-γ protein levels in the vitiligo skin (Figure 1E). These observations indicate that we successfully established mouse vitiligo mimicking human vitiligo immune signatures.

### 3.2. Altered Fibroblasts Set an Inflammatory Tone at the Stromal Site 

**Figure 2:**
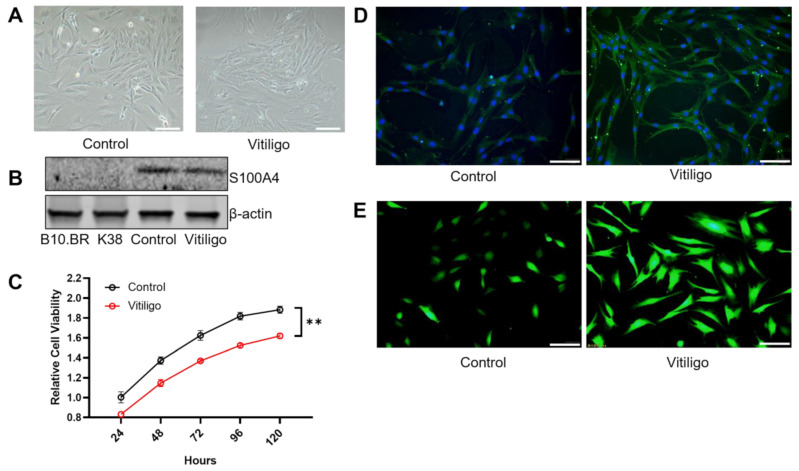
Mouse vitiligo fibroblasts recapitulate classical characteristics of inflamed human fibroblasts. (**A**) Representative phase−contrast images of fibroblasts isolated from control and vitiligo mouse lesions (*n* = 3). (**B**) Representative Western blots showed classical fibroblast marker S100A4 from fibroblasts in (**A**), but not immortalized melanocyte B10.BR and keratinocyte cell line K38 (*n* = 3). (**C**) Decreased CCK−8 cell viability from vitiligo fibroblasts in (**A**) (*n* = 3). (**D**) Representative immunofluorescent images showed higher DNA damage levels in vitiligo fibroblast (*n* = 3). (**E**) Representative immunofluorescent images showed increased ROS in vitiligo fibroblast (*n* = 3). Significant differences are indicated: two−tailed Student‘s *t*−test (mean ± SEM). ** *p* < 0.01. Scale bars = 100 μm (**A**,**D**,**E**).

Fibroblasts constitute a principal component of the skin structure, with mounting evidence indicating their role in inflammatory skin diseases. Previous studies suggest that fibroblasts are altered in vitiligo [8,24]. We isolated fibroblasts from both control and vitiligo mouse tails, both showing typical morphology (Figure 2A) and expressing classic fibroblast marker S100A4 (Figure 2B). We then performed a CCK-8 assay to measure their proliferation ability and found decreased fibroblast viability in vitiligo (Figure 2C). Human vitiligo fibroblasts have been closely linked to increased oxidative stress [24], leading to unavoidable DNA damage. We examined our mouse fibroblasts derived from vitiligo lesions and found DNA damage levels and increased ROS (Figure 2D,E) compared with the control. Thus, consistent with previous findings, our fibroblasts derived from mouse vitiligo lesions exhibit alterations prone to an overactive oxidative status that is destructive to fibroblasts and melanocytes.

### 3.3. Vitiligo Fibroblasts Show a T Cell-Related Transcription Profile

**Figure 3:**
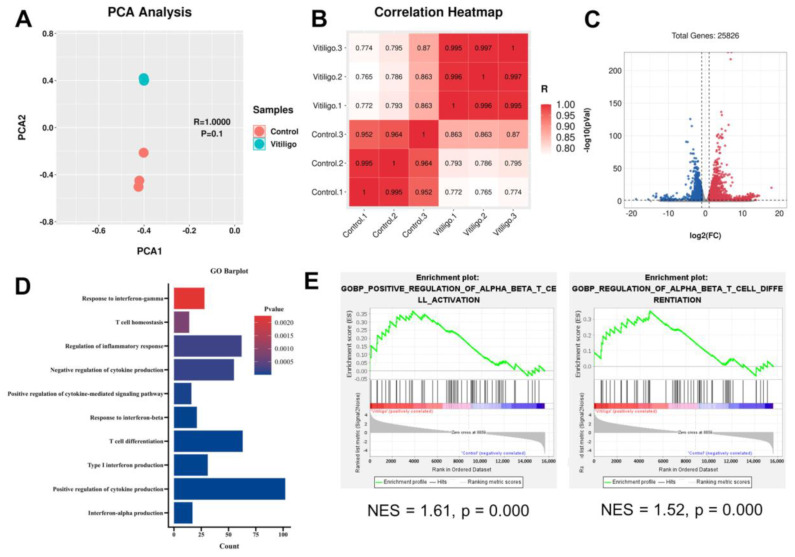
Vitiligo fibroblasts displayed a T cell−relevant inflammatory transcriptional profile. (**A**) PCA plots of RNA−seq data showed distinct control and IFN−γ−stimulated mouse skin fibroblast clusters. (**B**) Correlation heatmap of samples in (**B**). (**C**) Volcano plot of differentially expressed genes (DEGs) (blue, downregulated; red, upregulated). (**D**) GO barplot of DEGs−based enrichment of GO BP pathways. (**E**) GSEA enrichment plots of GO BP datasets calculated using full expression matrix. The text continues here.

Our mouse model highly resembled immune and stromal signatures of human vitiligo lesions, at least for T cell and fibroblast behaviors. Consequently, we sought to investigate fibroblasts further to understand better the stromal signals surrounding immune cells. Isolated mouse vitiligo fibroblasts were subjected to RNA sequencing. PCA plot demonstrated closely clustered samples within the same group (Figure 3A); the correlation heatmap also suggested the high consistency and reliability of our experiment (Figure 3B). Among 5473 differentially expressed genes, 3864 were upregulated and 1609 were downregulated (Figure 3C). To understand the biological functions, we performed an over-representation analysis of the Gene Ontology (GO) pathway using upregulated genes. In addition to interferon-related trials, we found significant enrichment of T cell-relevant biological processes, including T cell homeostasis and differentiation (Figure 3D). We then adopted GSEA and discovered enriched T cell activation and differentiation pathways (Figure 3E). In conclusion, our GO enrichment and GSEA results unveiled the previously unrecognized T cell regulatory potential of fibroblast in vitiligo.

### 3.4. IFN-γ Stimulates CCL2/CCL8 Expression in Fibroblasts through JAK2/STAT1 Signaling

**Figure 4:**
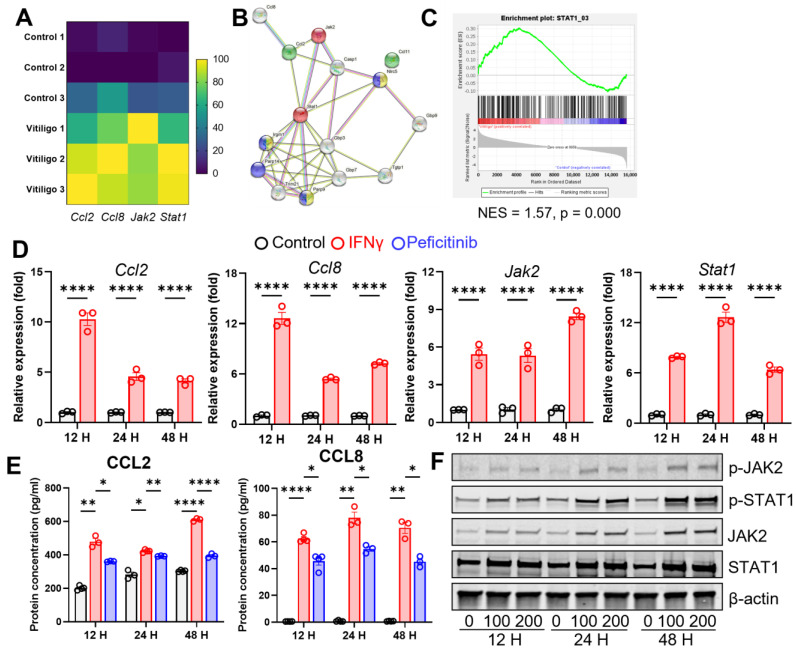
IFN−γ stimulates CCL2/CCL8 expression in fibroblasts through JAK2/STAT1 signaling. (**A**) Heatmap of selected genes in RNA−seq data from control and IFN−γ−stimulated mouse skin fibroblasts. (**B**) Genetic interaction mapping. (**C**) GSEA enrichment plots of transcriptional factor datasets calculated using full expression matrix. (**D**) qPCR of Ccl2, Ccl8, Jak2, and Stat1 showed elevated expression in fibroblasts stimulated with IFN−γ at different time points (12 h, 24 h, and 48 h) (*n* = 3). (**E**) ELISA quantification showed upregulated CCL2 and CCL8 in fibroblast cell line but not immortalized human melanocytes stimulated with IFN−γ at different time points (12 h, 24 h, and 48 h) (*n* = 3). (**F**) Representative Western blots showed elevated phosphorylated and total JAK2 and phosphorylated STAT1 in fibroblasts stimulated with IFN−γ at different time points (12 h, 24 h, and 48 h) (*n* = 3). Significant differences are indicated: 2−way ANOVA and the Šídák multiple comparisons test (mean ± SEM). * *p* < 0.05, ** *p* < 0.01, **** *p* < 0.0001.

Fibroblasts exert their roles in skin diseases through chemokine secretion [25,26,27]. We found drastically elevated chemokines CCL2 and CCL8 (Figure 4A). Moreover, functional protein interaction maps indicate that the JAK2-STAT1 regulatory network may govern such a process (Figure 4B,C). IFN-γ is the pivotal cytokine shaping a vitiligo-prone milieu. To verify our RNA-seq data, we stimulated normal fibroblast with IFN-γ to replicate the immune environment of vitiligo lesions. IFN-γ stimulation consistently raised Ccl2, Ccl8, Jak2, and Stat1 mRNA levels (Figure 4D). Uplifted CCL2 and CCL8 protein levels, along with the phosphorylation and total JAK2 and STAT1, were further determined by ELISA and Western blot, respectively (Figure 4E,F). Adding the JAK2 inhibitor peficitinib into the IFN-γ stimulation system abrogated the abovementioned phenomenon (Figure 4E). Indeed, in total, our data demonstrated that IFN-γ in the vitiligo lesions stimulates fibroblast’s expression of CCL2 and CCL8 through activating the JAK2/STAT1 signaling pathway.

### 3.5. CCL2 and CCL8 Skew the T Cell Population towards a Pathogenic Type 2 Profile

**Figure 5:**
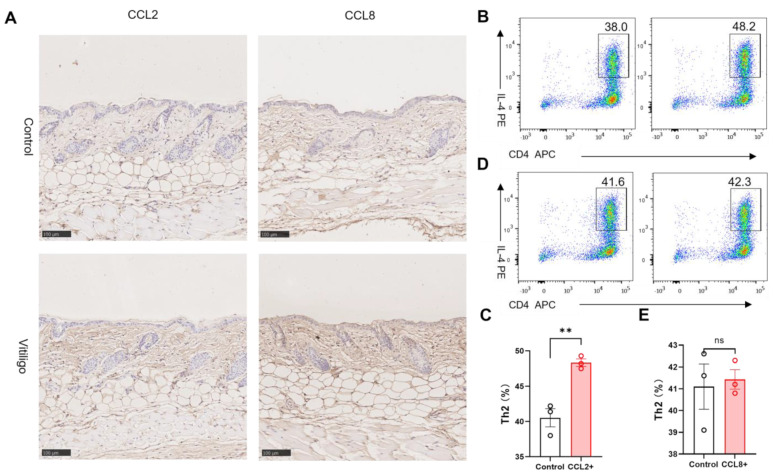
CCL2 promoted naïve CD4+ T cell polarization into Th2. (**A**) Representative immunohistochemistry images suggested upregulated CCL2 and CCL8 in vitiligo mouse skin (*n* = 3), scale bars = 100 μm. (**B**) Representative flow cytometric analysis showed elevated IL4+ T cells in the naïve T cell polarization system supplemented with CCL2 (*n* = 3). (**C**) Statistics of (**B**). (**D**) Representative flow cytometric analysis showed comparable IL4+ T cells in the naïve T cell polarization system supplemented with CCL8 (*n* = 3). (**E**) Statistics of (**D**). Significant differences are indicated: two−tailed Student‘s *t*−test (mean ± SEM). ** *p* < 0.01, ns no significance.

We further verified CCL2 and CCL8 protein levels in our mouse model and found a significant elevation of both cytokines (Figure 5A). As the IFN-γ stimulated vitiligo fibroblasts displayed a T cell-regulatory transcription profile (Figure 3D,E), we suspected that CCL2 and CCL8 might function through reshaping the T cell landscape. Previous reports suggested CCL2 and CCL8 involvement in Th2 polarization and homing, respectively [28,29,30]. Though vitiligo is dominated by type 1 immune response, recent studies also suggest the active involvement of the type 2 immune response [16]. We confirmed the drastic elevation of IL-4 in vitiligo mouse skin compared with the control (Figure 1E). As such, we performed T cell differentiation with or without the participation of CCL2. We found an increased Th2 proportion after CCL2 supplementation (Figure 5B,C). CCL8 has not been implicated in Th2 differentiation (Figure 5D,E), yet it is believed to induce the migration of a population of activated, differentiated Th2 cells [30]. Therefore, we summarize that CCL2 promotes type 2 T cell differentiation while CCL8 may recruit circulating or locally differentiated type 2 T cells. Overall, CCL2 and CCL8 play reciprocal roles in creating a type 2 cytokine profile in skin lesions.

## 4. Discussion

Our study discovered an IFN-γ-stimulated stromal signal driving type 2 T cell homing and differentiation. We showed that during vitiligo, pathogenic T cells secret IFN-γ not only to destroy melanocytes directly but also to direct fibroblasts to display a type 2 skewing profile. Mechanistically, in response to IFN-γ stimulation, the JAK/STAT signaling is activated to elevate CCL2 and CCL8 expressions. CCL2 promoted naïve T cell polarization into Th2 cells, and CCL8 is reported to attract Th2 cells. Luckily, such a pathological loop can be manipulated by a JAK inhibitor. However, only three biological replicates were subjected to RNA-seq in our experiment, resulting in lower statistical power. Nevertheless, we verified protein expression levels through IHC staining of more samples. It should also be noted that the transient CD4 depletion in the model, intending to suppress the Treg immunosuppressive effects, results in a short period in which almost all CD4 cells are absent, which may not reflect the nature of the disease. In short, our findings identified complex immune crosstalk and devised a strategy against it.

Even though melanocytes and T cells are indispensable participants in vitiligo [31], stromal signals from dermal cells may account for the complicated pathogenesis [8]. Fibroblast, a major constituent of the dermis, is no longer a passive element but a confederate of the immune system [9]. Through paracrine signaling, including cytokine and chemokine secretion, fibroblasts contribute to skin diseases such as psoriasis [4] and atopic dermatitis [5,32]. In vitiligo, researchers found that dermal fibroblasts can regulate skin pigmentation [33,34,35]. However, fibroblasts can also directly communicate with immune cells. One example is the recently published result showing fibroblasts are necessary to recruit and activate IFN-γ-producing CD8+ cytotoxic T cells through CXCL9/CXCL10 [3,36,37]. Nevertheless, studies reporting stromal–immune crosstalk remain few.

Chemokines such as CCL2 and CCL8 have been linked to immune cells [38]. Initially identified as monocyte chemoattractant proteins, their attraction role on monocytes has been well established [39,40,41,42]. In the context of vitiligo, we focused on its effects on T cells, the powerful force shaping the skin’s immune environment. CCL2-deficient mice consistently show defective Th2 response in different settings [28,43]. Our finding that CCL2 promoted Th2 polarization is in line with previous reports. There are CCL8 is structurally related to CCL2 yet displays different properties [44]. In mouse skin, CCL8 binds to CCR8 but not CCR2, providing a theoretical explanation for its unique role [30]. The evidence that the CCL8-CCR8 chemokine axis is a crucial regulator of the Th2 cell homing [30] also strengthens our speculation that CCL2 and CCL8 work synergistically to promote a type 2 profile bias at the stromal–immune interface. In this study, we only observed CCL2-induced Th2 skewing by flow detection of IL-4. It will be more convincing to monitor type 2 cells moving toward CCL8+ fibroblasts in vivo.

The observed type 2 attracting and promoting signals derived from fibroblasts can be pathogenic as most recently, researchers found both prominent type 1 and 2 immune responses in vitiligo lesions [16]. Thus, a strategy to inhibit such signaling may serve as a therapeutic approach. In our study, the underlying mechanism for CCL2 and CCL8 upregulation in response to IFN-γ turns out to be the JAK/STAT pathway. The JAK/STAT pathway is central for directing inflammatory cytokines [45]. Indeed, JAK inhibitor peficitinib successfully tamed CCL2 and CCL8 expression after IFN-γ stimulation. Currently, JAK inhibitors such as ruxolitinib, tofacitinib, and baricitinib are under active development for vitiligo and have shown strong efficacy [18]. Our proposed mechanism provided an extended rationale for the use of JAK inhibitors in vitiligo.

Here, we reveal that fibroblasts’ secretome in the dermal compartment skews the skin towards a type 2 cytokine profile, adding valuable insight into vitiligo stromal–immune interaction. We also identified JAK inhibitors as a therapeutic approach by deciphering the complex cytokine network. However, this project does not explore whether other factors expressed by irritated fibroblasts affect other immune cells. Further research may free us from a restricted view of melanocytes, highlighting other cutaneous cell populations in vitiligo.

## Data Availability

The analyzed data sets generated during the present study are available from the corresponding author upon reasonable request.
